# Dr. Hamell’s Address

**Published:** 1853-07

**Authors:** 


### Dr. HAMELL'S ADDRESS

*On Irregularity of the Teeth. Read before the Mississippi Valley Association of Dental Surgeons, held in Cincinnati February* 18*th and* 19*th*, 1853.

At our last meeting, the duty of reading a paper before the society at its present meeting, was assigned me. I will now *endeavor* to discharge that duty, by presenting to your consideration a few practical thoughts upon irregularity of the teeth.

Nature, when allowed to progress in undisturbed tranquility, is able to meet all her demands, and will bring forth the man perfect in all his parts, and hold him up to our view, truly the noblest work of God. Disease has not made its highway through his system; but he walks abroad wielding a scepter of power which none dare challenge. But, alas! how few are suffered to grow up to manhood without experiencing disease in some one or more of its withering and blighting forms. The little infant upon its mothers knee is not free from it. The man of middle age is not free from it. Nor is the old and venerable suffered to lie down in peace unmolested by it. If we search throughout the whole family of man, we cannot find any that have not been bound by some one of the links of that lengthy chain of diseases to which he is heir.

"The house we live in," is made up of parts, and none of these can be subjected to disease or malformation without more or less affecting the whole. The teeth, those beautiful organs which give form to the face and expression to the countenance, constitute a prominent part—and is it possible that they can be so arranged as not to allow the jaws their proper motion for mastication, without producing deleterious effects? Certainly not. To us, then, who have studied the nature and cause of irregularity in these organs, suffering humanity turns and with beseeching looks whispers help.

The correction of irregularity of the teeth is among the first operations which the dentist is called upon to perform. It develops itself as the teeth are developed; and becomes permanent as the teeth approach maturity. We fear that many of those professing to be dentists, do not give this subject that amount of study and attention which its importance demands.

I am acquainted with a young gentleman whose teeth made their appearance through the gum very irregularly; he showed them to a dentist, who most ill-advisedly, extracted both of the lateral inscisors so as to give the other teeth room to arrange themselves. Another case that chanced to fall under my notice was where one of the central incisors had ing been extracted, because it chanced to sleep beneath the gum until its neighbor got the start of it. I need not comment upon these cases; you can judge concerning the propriety or impropriety of such practice yourselves. I give them merely to show how much men may err in judgment with respect to this matter, who have not made it a subject of close investigation.

Irregularity of the teeth is a forerunner of disease not only in themselves, but also in the surrounding parts. The teeth, by undue pressure, induce absorption of the gums and processes, or if these be strong enough to ressist this pressure then their enamel will be fractured, and thereby they will become fit subjects for decay.

Seeing then that these are some of the results of irregularity in the arrangement of these organs, how necessary is it that we should be armed with a correct judgment and a cunning hand, to enable us to restore erring nature to its proper place again. The benefits arising from regularly arranged teeth are numberless, the troubles arising from it are also numberless, and call forth our warmest sympathy.

Why is it that the agriculturist is so particular in planting his corn to have all the rows straight, and not more than four stalks in a hill? Is it not because experience and observation have taught him that thereby he will be enabled to keep the weeds from gaining the ascendency, and also that the ears will grow larger?

This same rule, then, which has proved itself good in the vegetable kingdom, will also hold good in the animal. If the teeth are regularly arranged, the person, in whose mouth they are situated, will be enabled to keep all particles of extraneous matter from being retained in contact with them, and they will more nearly approach that standard of perfection which they were designed to occupy. But if they are crowded so as to be thrown out of their places, or kept from coming through the gum at the appointed time, or the enamel prevented from being properly deposited upon their crowns, then the results can be better anticipated than expressed. Just the thing to be done in such cases as this, is often very difficult to decide; but one thing is cerain, temporizing half-way work will do no good. We think there is no speciality of dental practice that draws so heavily upon our judgment as the correction of irregularity of the teeth. If a tooth is to be extracted for the purpose of gaining room for the others, how important is it that we weigh well the matter, with regard to which tooth we will extract, before we proceed to action. We have a fair specimen of this off-hand kind of work, in the examples already mentioned.

The tooth occupying the wrong place, is not, in nine cases out of ten, the one that ought to be extracted; but one or more in the back part of the mouth, although they may occupy their proper places, must be sacrificed, that those in front, and which will more decidedly retain the contour of the face, may be brought into their proper places.

The time of life, as laid down in the best dental works for the performance of this operation, is prior to the sixteenth year, though this operation may be performed after this time, yet with far less chances of success. At this time, the teeth having all made their appearance through the gum, except the wisdom teeth, the judicious dentist is able to determine whether his services are really required or not; and if required he should at once adopt such means as will but correct the fault. It would not be proper for me to occupy your time by an enumeration of all the particular appliances that have been employed for this purpose. If the general subject has been well studied, the peculiarities of each case will suggest to our minds that which will best accomplish the desired end.

Although the time we have mentioned is the proper one for correcting irregularity, yet a majority of those who make application for this purpose, defer it until they have arrived at their eighteenth or nineteenth year. And here a difficulty interposes, which is here to be disposed of satisfactorily to all parties. Who is to bear the blame for this delay of application? Is it the dentist for not placing the means of information on this subject in the hands of his patrons? Or is it these persons themselves for not informing themselves on the subject? We are rather inclined to give the dentist a balance, but not overmuch.

We apprehend that the sole cause in a majority of these long-standing cases of irregularity, is the want of information on the subject, on the part of those having the care of children. Some may argue that indulgence is the cause. We admit that in many cases it has something to do in the matter; but we think ignorance is the cause of this also, and that both would be set aside, as the importance of this matter were fully made clear. But how is this reformation to be brought about? I know that many of us are inclined to console ourselves; and say to our patients when they apply too late to have anything done, "You should have had this matter attended to sooner, without trying to devise means that will prevent others from falling into the same error."

We think the most effectual way to eradicate this evil, would be for every dentist to provide himself with an essay on all points of dental practice, but especially upon irregularity of the teeth—have it put up in pamphlet form—and give a copy to every person that came into his office. By so doing, he would not only use a means that would tend most powerfully to eradicate an evil which has so long enslaved community, but also free himself from all responsibility on this subject.

